# Beeldzorg in de gehandicaptenzorg – door corona eindelijk een doorbraak! Of toch niet?

**DOI:** 10.1007/s12508-022-00352-z

**Published:** 2022-05-31

**Authors:** Marko Mravunac, Sanne van der Weegen, Agnes van der Poel

**Affiliations:** 1 Vilans, Utrecht, Nederland; 2 Vilans, Utrecht, Nederland; 3 Academy Het Dorp, Arnhem, Nederland

## Abstract

Door corona hebben drie gehandicaptenzorgorganisaties versneld technologie voor beeldbellen en beeldzorg ingezet. Sinds 2021 wisselen zij daarover ervaringen uit in de Innovatie-impuls Gehandicaptenzorg. We inventariseerden hoe begeleiders, cliënten en naasten beeldzorg ervaren. Uit de interviews blijkt dat ze blij zijn met de mogelijkheden die beeldzorg biedt, maar ook dat ze het vooral zien als back-up. Voor duurzame implementatie van beeldzorg is een visie nodig op de toekomst van de zorg en de rol die digitale zorg daarin speelt. Deze resultaten zijn daar behulpzaam bij.

## Abstract (en)

Covid-19 accelerated the implementation of digital care in three organisations in disability care. From 2021, they exchanged experiences in the program ‘Innovatie-impuls Gehandicaptenzorg’. We examined how professionals, clients and family experienced digital care. The interviews showed that they are happy with the possibilities that digital care offer, but also that they mainly see it as back-up. For sustainable implementation of digital care, a vision is needed on the future of care and the role of digital care therein—these results are helpful for that.

## Corona en beeldzorg

In maart 2020 bereikte het coronavirus ook ons land en was een lockdown onvermijdelijk. Er waren bezoekregels en werken vanuit huis werd de norm. Afspraken en contact gingen via beeldbelapplicaties als Teams of Zoom. Professionals in de gehandicaptenzorg moesten ook zo veel mogelijk vanuit huis werken, waardoor het contact met collega’s en ambulante cliënten via beeldbellen verliep. In de gehandicaptenzorg werd al wat langer gewerkt met beeldbellen en beeldzorg: uit de eHealth-monitor 2017 blijkt dat 27 % van de begeleiders van mensen met een verstandelijke beperking aangeeft dat ze zelf of hun collega’s in de instelling gebruik hebben gemaakt van beeldbellen [1]. Van grootschalige implementatie en borging in de sector blijkt geen sprake. Door de lockdown leken zorg en ondersteuning op afstand via beeldbellen opeens wél te kunnen en een enorme vlucht te nemen [2, 3]. ICT-afdelingen draaiden op volle toeren. Er werd geïmproviseerd, zodat intramurale cliënten toch hun naasten konden zien en spreken. En zodat zorgmedewerkers toch via beeld contact hadden met hun ambulante cliënten. In juni 2020 kon de wereld voorzichtig weer open dankzij de beschikbaarheid van tests en mocht er meer, om in oktober weer in een gedeeltelijke lockdown te belanden. Er werd voornamelijk thuisgewerkt, maar het volledige isolement kwam niet terug. Iedereen mocht drie personen per dag thuis ontvangen. Dit zorgde ervoor dat de zorg aan ambulante cliënten face-to-face door kon blijven gaan en dat familie hun naasten op de woongroepen wel (met registratie en mondkapje) mochten bezoeken.

In de gehandicaptenzorg loopt sinds najaar 2019 de Innovatie-impuls Gehandicaptenzorg, een VWS-programma uitgevoerd door Vilans en Academy Het Dorp, waarin bijna dertig zorgorganisaties ondersteuning krijgen bij de duurzame implementatie van technologie die bijdraagt aan een zorginhoudelijk vraagstuk [4]. Binnen de Innovatie-impuls zijn zes zorgorganisaties het themanetwerk *Corona en contact op afstand* gestart, omdat ze tijdens corona *versneld* een technologie voor ‘contact op afstand’ hadden ingezet. In het themanetwerk wisselden ze ervaringen uit en gingen ze aan de slag met duurzame implementatie. Drie van deze zes organisaties hebben tijdens de coronapandemie beeldzorg ingezet voor ambulante begeleiding en/of dagbesteding. Om te achterhalen hoe de implementatie is gegaan en wat de ervaringen en verwachtingen van cliënten, zorgmedewerkers en naasten zijn, voerden we een verkenning uit. We zochten antwoord op de volgende vragen: hoe is de implementatie gegaan? Hoe hebben medewerkers en cliënten beeldzorg ervaren? Willen ze beeldzorg blijven gebruiken en waarvoor? Voor zover we weten, is een dergelijke inventarisatie waarin het cliënt- en medewerkerperspectief centraal staat nog niet eerder in de gehandicaptenzorg uitgevoerd. De zorgorganisaties gebruiken de resultaten om hun implementatieactiviteiten verder te plannen en verbeteren. En de opgedane inzichten kunnen andere gehandicaptenzorgorganisaties helpen bij de keuzen rond beeldzorg.

## Interviews

Om deze vragen te beantwoorden hielden we semigestructureerde interviews met cliënten, naasten en begeleiders. We hebben samen met de projectleiders van de zorgorganisaties deelnemers geworven. De topiclist bevatte vragen over 1) de implementatie, 2) ervaringen en 3) verwachtingen over toekomstig gebruik van de tijdens de coronapandemie ingezette technologie voor beeldzorg. Alle interviews werden via een beeldbelapplicatie (Teams, Zoom of Mobiléa) afgenomen. Van februari tot en met juli 2021 zijn negen cliënten, tien medewerkers en twee naasten geïnterviewd (zie tab. 1). Bij organisaties A en B werden de interviews tijdens de lockdown gehouden, bij organisatie C gebeurde dat tijdens de periode waarin versoepelde maatregelen golden. Als een cliënt dat wilde, was een persoonlijk begeleider aanwezig. Cliënten hebben een licht verstandelijke beperking, autisme en/of ggz-problematiek. De interviews zijn getranscribeerd en geanalyseerd. De geïnterviewden gaven informed consent.

**Table 1:** 

	organisatie		
	A	B	C
*technologie*	Mobiléa-beeldbeloplossing voor de zorg	Teams, WhatsApp	Teams, WhatsApp
*beeldzorg*	medewerker-cliëntcontact; dagbesteding	medewerker-cliëntcontact	medewerker-cliëntcontact
*interviews*	4 cliënten	3 cliënten	2 cliënten
	3 ambulant begeleiders en 1 begeleider dagbesteding	3 ambulant begeleiders	3 ambulant begeleiders
	–	1 naaste	1 naaste

## Hoe verliep de implementatie?

Binnen de drie organisaties is de implementatie op verschillende manieren verlopen. Bij organisatie A gaf de leverancier een 3 uur durende training over Mobiléa aan medewerkers van één locatie. Collega’s van andere locaties kregen een-op-een uitleg van de projectleider of een collega die de training had gevolgd. Cliënten kregen uitleg van hun begeleider.

Bij organisatie B kregen medewerkers een mail met een korte uitleg en de mogelijkheid om een training te volgen over het gebruik van Teams. Voor cliënten konden ze een tablet aanvragen. Andere cliënten gebruikten hun smartphone. Op verzoek van cliënten is ook WhatsApp gebruikt.

Bij organisatie C kregen medewerkers een e‑mail en een presentatie over het gebruik van Teams. Teams was in eerste instantie bedoeld voor contact tussen medewerkers. Cliënten vonden het gebruik van Teams te ingewikkeld en stapten over op WhatsApp.

Over de privacyrisico’s van WhatsApp is door de organisaties veel gesproken. Uitkomst was dat de AVG geen reden is om deze app niet te gebruiken, mits deze app niet gebruikt wordt voor persoonlijke, gevoelige informatie [5].

Veel begeleiders geven aan dat ze weinig moeite hadden met het leren gebruiken van de technologie, omdat ze nieuwsgierig waren. Voor hun collega’s die wat minder digivaardig zijn, zou uitgebreide ondersteuning nodig zijn. Meer bekendheid over en met de technologie zou de slagingskans vergroten.

Wil Mobiléa bij ons een succes worden, dan moet er eerst bekendheid worden vergaard bij de medewerkers. Ik heb het geleerd van mijn collega’s, maar andere collega’s weten niet dat het bestaat. – begeleider

Wat mij betreft mag de organisatie meer het voortouw nemen en ons aan de hand nemen om collega’s te enthousiasmeren. Ja, over de manieren waarop beeldbellen echt een meerwaarde kan creëren, ook in de ambulante hulpverlening. […] Ik ben er wel enthousiast over, maar ik weet niet wat de mogelijkheden zijn. – begeleider

Teams is wel echt lastig voor cliënten. Dan was er geen link, of moesten ze een nieuw account maken of een nieuw wachtwoord maken. En: ‘Ik kan er niet inkomen’. – begeleider

## Wat zijn de ervaringen?

### Live contact en huisbezoeken versus beeldbellen

Begeleiders en cliënten geven aan dat ze blij waren dat beeldbellen en beeldzorg tijdens de lockdown mogelijk waren. Het merendeel verkiest echter fysieke zorg en real-life contact. Begeleiders lichten toe dat ze dan beter kunnen inschatten hoe het emotioneel en sociaal met de cliënt gaat en wat er nodig is. Voor medewerkers is het ook lastig om via beeldbellen de staat en leefomgeving van cliënten thuis in te schatten. De geur van het huis, of er vuile vaat staat, of het opgeruimd is, bijvoorbeeld, zijn indicaties van hoe het met de cliënt gaat. Ook vinden ze het lastig om via beeld met cliënten over hun problemen te spreken.

Beeldbellen is fijn, maar elkaar in het echt zien is beter. – cliënt

Een van mijn doelen is altijd: kijken hoe het in de huishouding gaat, mijn ervaring is met beeldbellen dat ze dan de keuken net niet laten zien. Tenzij ik ernaar vraag. En dan krijg ik een hoekje te zien en dan moet ik echt doorvragen en dan voel ik mij meer een politieagent. Terwijl als ik binnen kom, dan scan ik en dan weet ik meestal gelijk: oké, ze zit in dat humeur, dus ik moet die kant uit. En dat vind ik het lastigste van het beeldbellen. – begeleider

Ik ben er zelf niet zo van. Ik vind face-to-face in werkelijkheid veel fijner. Maar als het echt niet anders kan agendatechnisch of het is zo’n kort gesprek, dan vind ik het wel fijn. – naaste

Beeldbellen over praktische zaken is handig met cliënten met een wat hoger niveau, maar een van mijn doelen is meestal ook wel: hoe zit iemand in z’n vel? Vaak zijn wel complexe problemen en dat gaan zij niet vertellen via beeldbellen. Of ze vertellen te weinig. Dat is lastig om daar dan structuur in te geven. – begeleider

Ja, dat is bij mij wel zo, dat ik mijn gevoel even kan uitschakelen en dan zet ik een masker op. Dan wil ik niet laten merken dat er iets is. Als zij [begeleider] hier is, dan ziet ze alles meteen. – cliënt

### Telefonisch contact versus beeldbellen

Medewerkers geven aan dat zij – net als voor corona – tussen de consulten (geplande momenten/huisbezoeken) door geregeld gebeld worden door ambulante cliënten. De meeste medewerkers zijn van mening dat beeldbellen waardevoller is dan telefonische gesprekken. Daarbij merken ze op dat beeldbellen niet altijd spontaan kan vanwege privacy, als ze bijvoorbeeld bij een andere cliënt in huis (of in hun eigen huis) zijn. Ook cliënten vinden bellen met beeld fijn.

Ik zie zelf wel echt een meerwaarde als aanvulling op wat wij bieden. Ik heb een cliënt die belt toch zeker drie keer per week. Daar kan ik aan de telefoon slecht polsen of hij begrepen heeft wat ik bedoel. Met beeldbellen kan ik dan beter zien of hij het begrepen heeft. Als je dan beeldbelt, en ziet, dan kan ik veel aflezen van de non-verbale communicatie of diegene het heeft begrepen. – begeleider

Dit is beter dan gewoon bellen. – cliënt

### Beeldbellen is efficiënt voor praktische zaken

Begeleiders beoordelen de laagdrempeligheid van beeldbellen als positief. Praktische zaken kunnen gemakkelijk via beeld afgehandeld worden, en dat levert ook tijdswinst op omdat er geen reistijd is. Cliënten ervaren bijvoorbeeld vaak grote stress als er een brief binnenkomt van een instantie als de gemeente. Nu laten cliënten de brief via beeld zien, en dan is het vaak al voldoende als de begeleider aangeeft dat het beantwoorden van de brief kan wachten tot het volgende live consult. Daarnaast ervaren medewerkers dat het makkelijker is om een gesprek met meerdere mensen, of met mensen ver weg, te plannen met beeldcontact. Nu haakt een begeleider ook vaker aan bij gesprekken met bijvoorbeeld een arts of andere hulpverleners.

Mooi om te zien hoe makkelijk dat kan en hoeveel tijd me dat scheelt qua belletjes. – begeleider

Ik geloof echt dat een gedeelte van de zorg overgenomen kan worden door beeldzorg. Als een cliënt bijvoorbeeld een brief heeft die die niet begrijpt, dan moet ik normaal een kwartier reizen […]. Met beeldbellen laat de cliënt de brief zien via het scherm en ik kan meteen uitleggen hoe en wat, dan scheelt me dat een kwartier heen en terug. En ik denk dat er meer gevallen zijn waarin je zo kan werken. – begeleider

Als je dingen op een computer moet regelen, dat moet je gewoon live doen, anders zit je met toestemmingen en DigiD, et cetera. Maar als het gaat over de post of voor de kleine dingen, dat kan je via beeldbellen checken. – begeleider

Ik vind het wel prettig om bijvoorbeeld een foto van een rekening te sturen via de chat. Ik vind het wel fijn om tussendoor te kunnen vragen. – cliënt

We zaten daar [specialist in ziekenhuis] 10 minuten voor een uitslag en die was negatief. Dat had makkelijk via beeldbellen gekund. Dat zou ook nog € 40 schelen. Thuis ben je ook meer op je gemak. – cliënt

Bijvoorbeeld als een cliënt naar de dokter moet en je zou eigenlijk mee willen maar dat kan niet agendatechnisch. Maar dat je dan via beeldbellen met de cliënt en de arts wel kan aanhaken. En dit geldt voor heel veel andere hulpverleners. – begeleider

## Wat zijn de verwachtingen over toekomstig gebruik?

De meeste geïnterviewden van de drie zorgorganisaties betwijfelen of ze beeldzorg in dezelfde mate blijven gebruiken als er geen coronamaatregelen van kracht zijn. Cliënten, naasten en de meeste begeleiders geven aan dat ze beeldzorg voor medewerker-cliëntcontact sinds de versoepelingen nog maar minimaal gebruiken. Volgens hen is beeldbellen een fijne oplossing als het echt niet anders kan tijdens een lockdown of beperkende maatregelen, of als iemand in quarantaine zit.

Als dingen straks weer normaal worden, zou ik het wel minder gebruiken omdat ik het minder nodig heb. […] Ik denk dat het wel een heel mooi middel is voor dagbesteding. Dus als mensen komen, dan is het niet nodig. Maar als ze niet kunnen komen, door vervoer of wat dan ook, dan kan iemand nu wel online vanuit huis meedoen. Dat was eerst niet zo. Dus ik zou het wel weer gebruiken als het weer nodig zou zijn. – begeleider

Omdat nu na de corona alles mag, is het fijner om dingen samen te regelen dan via beeldbellen. Maar als je niet kan komen, dan is het wel makkelijk dat het met beeldbellen kan – cliënt

Ik weet niet of ik na corona Mobiléa blijf gebruiken, wij zijn wel van het contact met de cliënt. Maar misschien kan het handig zijn. […] Ik denk dat die cliënt Mobiléa wel op zijn tablet laat staan, maar ze zijn wel toe aan fysiek contact ook, dus het gebruik van Mobiléa wordt denk ik minder na corona. – begeleider

Toen mochten we weer langskomen. Vond ik fijner dan beeldbellen, want dan kan je elkaar nog beter zien. [*Wat zou je vinden van een combinatie van beeldbellen en langskomen?*] Zou ik niet erg vinden. Maar soms als je iets niet snapt, dan is het soms lastig en is het fijn als je effe langs kan komen. – cliënt

Eén begeleider is uitgesproken positief over beeldzorg:

Ik zou het fysieke bezoek willen inkorten en dan een standaard beeldbelmoment inplannen. In dezelfde tijd is er dan vaker contact, ja, en dat kan stress bij de cliënt verlagen. En het scheelt misschien tussentijdse belletjes. – begeleider

## Conclusie

Door de coronapandemie nam de beeldzorg een vlucht, maar ze lijkt niet te beklijven. De implementatie tijdens de coronapandemie lijkt onvoldoende systematisch aangepakt. Medewerkers en cliënten (en naasten) grijpen snel terug op het oude gedrag. Cliënten en medewerkers geven aan blij te zijn dat beeldbellen en beeldzorg tijdens de lockdown sinds maart 2020 mogelijk waren voor ambulante begeleiding en dagbesteding. Het was via beeld of niet. Beeldbellen heeft in vergelijking met telefonisch contact het voordeel dat je gezichtsuitdrukkingen van elkaar kan zien, waardoor de medewerker beter kan inschatten hoe het met de cliënt gaat. Daarnaast hebben beeldbellen en beeldzorg een blijvende meerwaarde bij praktische zaken, zoals bij het beoordelen van een brief door de begeleider, en wordt het makkelijker om een cliënt bij te staan bij afspraken met andere hulpverleners. De meeste cliënten en begeleiders geven echter aan dat ze live contact prefereren en dat ze beeldzorg vooral gebruiken als ‘back-up’. Voor deze back-up wordt vaak WhatsApp gebruikt, waar privacyrisico’s aan verbonden zijn.

## Hoe verder?

Net als in de huisartsen-, verpleeg- en ziekenhuiszorg, willen organisaties in de gehandicaptenzorg duurzaam gebruikmaken van beeldzorg, ook omwille van de arbeidsmarktproblematiek [6–8]. Naast aandacht voor factoren die implementatie beïnvloeden zullen zorgorganisaties – en in het bijzonder de gehandicaptensector als geheel – een nieuwe manier van werken moeten ontwikkelen en hierop moeten sturen vanuit beleid. Samenwerking tussen organisaties en een heldere en gedragen visie op de toekomst van de zorg en de rol van digitale zorg en contact daarin zijn noodzakelijk. Bestuur, managers, medewerkers, cliënten en naasten moeten samen verkennen hoe die andere manier van zorg eruit kan zien. Aandacht voor privacy in het kader van de AVG moet daar onderdeel van zijn. Visies en implementaties in andere sectoren kunnen inspireren, net als bijvoorbeeld een initiatief als Digicontact in de gehandicaptenzorg [9].
